# 

**Published:** 1818-05

**Authors:** 


### CATALOGUE OF MEDICAL BOOKS.

Facts and Observations on Liver Complaints, and those various and extensive Derangements of the Constitution arising from Hepatic Obstruction; with practical Remarks on the Biliary and Gastric Secretions, and upon other important Points essential to Health; pointing out a successful Mode of Treatment, illustrated by numerous Cases. The third Edition, very considerably enlarged. By John Faithhorn, formerly Surgeon in the E. I. C.'s Service.

Modern Maladies, and the present State of Medicine.—The Anniversary Oration, delivered March 9, 1818, before the Medical Society of London. By D. Uwins, M.D. Member of the London College of Physicians, and one of the Secretaries of the Society. Published at the request of the Society. 8vo.

The Influence of Tropical Diseases on European Constitution: to which is added, Tropical Hygiene, or the Preservation of Health in all Hot Climates; adapted to general perusal. By Jas. Johnson, M.D. Second edition, greatly enlarged. 8vo. 15s.

General Views relating to the Stomach, its Fabric, and Functions. By T. C. Speer, M.D. 8vo. 7s. 6d.

A Treatise, which obtained the Prize on this Question—"What are the Symptoms which indicate, or contra-indicate, Blood-letting in Fevers, whether intermittent or continued, designated under the terms Putrid, or Adynamic, Malignant, or Ataxic?" proposed by the late Academical Society of Paris, for the Meeting of 1812. By J. van Rotterdam. Translated from the French, by J. Taylor, M.D. 8vo. 10s.

Observations on the Treatment of the Epiphora, or Watery Eye; and on the Fistula Lacrymalis: together with Remarks on the Introduction of the Male Catheter, and on the Treatment of Hæmorrhoids. To which are now added, Observations on the Near and Distant Sights of different Persons, &c. By the late James Ware, F.R.S. &c. Edited by his Son, Martin Ware. New edition. 8vo. 8s.

---

### *NOTICES TO CORRESPONDENTS.*

*Communications are received from* Dr. Kinglake, Dr. Wood, Dr. Parkinson, Dr. Adams; Messrs. Littleton, Edwards, M'Turk, Brown, *and* Perry; N. R. S. *and other anonymous writers.*

